# Beyond the darkness: recent lessons from etiolation and de-etiolation studies

**DOI:** 10.1093/jxb/erz496

**Published:** 2019-12-19

**Authors:** Tegan Armarego-Marriott, Omar Sandoval-Ibañez, Łucja Kowalewska

**Affiliations:** 1 Max Planck Institute of Molecular Plant Physiology, Potsdam, Germany; 2 Faculty of Biology, Department of Plant Anatomy and Cytology, University of Warsaw, Warszawa, Poland; 3 University of Essex, UK

**Keywords:** chloroplast biogenesis, de-etiolation, etiolation, etioplast, prolamellar body, skotomorphogenesis

## Abstract

The state of etiolation is generally defined by the presence of non-green plastids (etioplasts) in plant tissues that would normally contain chloroplasts. In the commonly used dark-grown seedling system, etiolation is coupled with a type of growth called skotomorphogenesis. Upon illumination, de-etiolation occurs, marked by the transition from etioplast to chloroplast, and, at the seedling level, a switch to photomorphogenic growth. Etiolation and de-etiolation systems are therefore important for understanding both the acquisition of photosynthetic capacity during chloroplast biogenesis and plant responses to light—the most relevant signal in the life and growth of the organism. In this review, we discuss recent discoveries (within the past 2–3 years) in the field of etiolation and de-etiolation, with a particular focus on post-transcriptional processes and ultrastructural changes. We further discuss ambiguities in definitions of the term ‘etiolation’, and benefits and biases of common etiolation/de-etiolation systems. Finally, we raise several open questions and future research possibilities.

## Teaser

A discussion of recent discoveries in the etiolation/de-etiolation field, focusing on post-transcriptional processes and ultrastructural changes, along with comments on usage of the term ‘etiolation’, and of common etiolation/de-etiolation systems.

## Introduction: defining etiolation

**Fig. 1.**
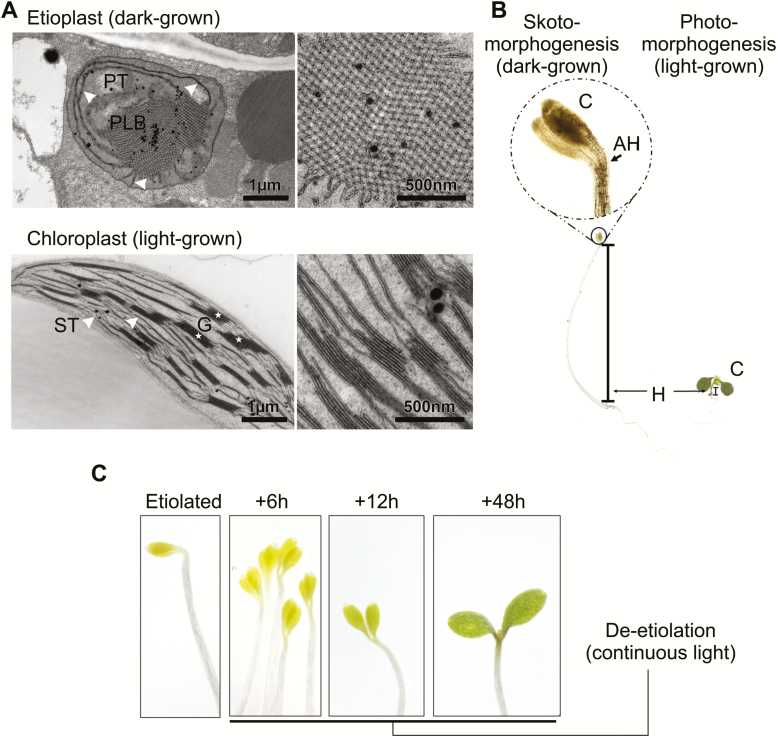
Etiolated phenotypes in plants (exemplified in Arabidopsis). (A) Plants grown in extended darkness develop etioplasts (upper panels). These plastids are physically defined by the presence of a paracrystalline membrane structure called prolamellar body (PLB), as well as prothylakoids (PT, indicated by white arrowheads). In the light, photosynthetic tissue develops chloroplasts (lower panels), which are defined structurally by thylakoid membranes that contain grana stacks (G, white asterisks) and stroma lamellae called stroma thylakoids (ST, white arrowheads). Images are from 6-day-old dark-grown Arabidopsis plant (upper panel), and a light-grown Arabidopsis plant at the rosette stage (lower panel). (B) Etiolation and de-etiolation studies generally involve germination and growth of seedlings in darkness, resulting in skotomorphogenic growth (left). This is defined by the presence of an apical hook (AH), closed and pale cotyledons, and an elongated hypocotyl. By contrast, plants grown the light (photomorphogenic conditions; right) have shorter hypocotyls, and open, green cotyledons. C, cotyledon; H, hypocotyls. Images taken from a 7-day-old dark-grown and a 9-day-old light-grown Arabidopsis seedling. (C) De-etiolation of dark-grown (etiolated) seedlings involves straightening of the apical hook, opening and greening of the cotyledons, as well as the transition from etioplast to chloroplasts (refer to Fig. 3). The etiolated seedlings were exposed to continuous white light (95 µmol photons m^−2^ s^−1^) for 6, 12, and 48 h.

Etiolation involves prolonged growth in the absence of light that results in the development of etioplasts in tissue that would have chloroplasts if subjected to light. Etioplasts do not contain chlorophyll or stacked thylakoid membranes, but rather have a paracrystalline lipid–pigment–protein structure known as the prolamellar body (PLB). The PLB consists largely of the plastid lipids monogalactosyldiacylglycerol (MGDG) and digalactosyldiacylglycerol (DGDG), and an association of the chlorophyll precursor protochlorophyllide (Pchlide), the light-dependent protochlorophyllide oxidoreductase (LPOR) that is responsible for its conversion, and the cofactor NADPH (Fig. 1; etioplast composition and structure reviewed, for example, in Kowalewska *et al.* (2019) and Pribil *et al.* (2014)).

As most scientifically observed etiolation systems involve (aseptic) germination and growth of seedlings in complete darkness, the term ‘etiolated’ is commonly defined additionally by the presence of a skotomorphogenic phenotype of elongated hypocotyls, shortened roots, and small, closed cotyledons (Fig. 1; reviewed in Josse and Halliday, 2008). In these systems, the light-driven etioplast-to-chloroplast transition is coupled to a transition from skotomorphogenic to photomorphogenic growth. These morphogenic traits are often portrayed in quantifiable and continuous terms, with variables of hypocotyl length, apical hook angle, and cotyledon angle considered. By these definitions, aberrant ‘photomorphogenic in darkness’ or ‘skotomorphogenic in light’ phenotypes have been utilized to identify multiple components involved in light sensing, signaling, or downstream responses. Many of these components have since been shown to have broad roles in non-etiolation-related light response.

The majority of the data discussed in this Expert View refer to work undertaken in such seedling-based etiolation/de-etiolation systems. The various limitation of these systems and possible alternative or complementary systems are also discussed (in the section ‘New systems required and new lessons learned’).

More broadly, the term ‘etiolated’, which has etymological roots in the French *étiolier* (i.e. straw), is still used as a descriptor for a range of pale or yellowing phenotypes. These include nitrogen-deficient rice (*Oryza sativa*; Sun *et al.*, 2018*a*), graft-incompatible pomello (*Citrus grandis*; He *et al.*, 2018), and heavy-metal-treated wheat (*Triticum aestivum*; Semenova *et al.*, 2017). Similarly, a skotomorphogenic phenotype observed in infected light-grown creeping bentgrass (*Agrostis stolonifera*; Roberts *et al.*, 2016) was recently termed ‘bacterial etiolation’. We consider these phenotypes to be largely outside our personal definition of etiolated tissues (i.e. having etioplasts), and will not discuss them within this work. Nonetheless, we note that in recent years, similar ‘etiolated’ phenotypes have been linked to pigment accumulation (Chen *et al.*, 2018*b*) and light signaling defects (Peng *et al.*, 2019). Furthermore, the pale barley (*Hordeum vulgare* L.) *albostrians* mutant (Muramoto *et al.*, 1999), has been shown to contain structures in its albino sectors that are highly reminiscent of transforming PLBs (Li *et al.*, 2019). As such, these ‘etiolated’ plants should be consdered a potential source of new players in the regulation of chloroplast development, particularly in non-model species. Finally, this review will not discuss etiolation-like responses in non-angiosperm species, a still under-represented and debated research field (reviewed in Mathews, 2006).

### Recent developments in understanding etiolation and the etioplast-to-chloroplast transition

**Fig. 2.**
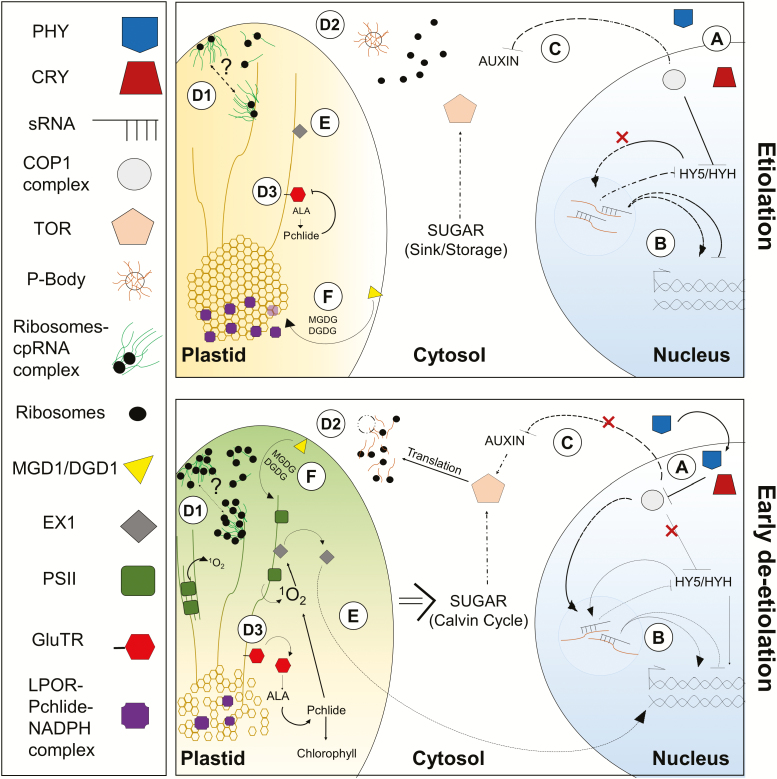
Signaling cascade and recently described players in de-etiolation. This simplified model shows a basic overview of (A) the PHY/CRY-mediated light-responsive signaling cascade, and (B–F) recent discoveries in the field discussed in this review. The upper panel shows the etiolated state, the lower panel shows the changes that occur early upon de-etiolation. (A) Light is perceived by photoreceptors such as phytochromes (PHY) and cryptochromes (CRY), resulting in indirect activation of the expression of *Elongated Hypocotyl5/HY5 Homolog (HY5/HYH)*-dependent photomorphogenesis-related genes by repression of the COP1 complex. (B) Small RNAs (sRNA) modulate transcript accumulation of both light-signaling molecules and ownstream effector genes, and the sRNA pathway itself is also controlled via light signaling pathways. (C) TOR indirectly activates translation via auxin, and is itself stimulated by light as well as by sugars. (D) Physical sequestration can limit functionality. (D1) Increased translation in the plastid is likely linked to increased ribosome density, as opposed to occupancy. (D2) Cytosolic transcripts are sequestered in processing bodies (P-bodies) during etiolation, with release allowing their translation. (D3) GluTR is soluble and active in the light, with the soluble form correlating with chlorophyll content during greening. (E) Retrograde signalling mediated by 1O2 produced by the early assembly of the oxygen evolving complex of PSII might contribute to the EXECUTER1 signaling pathway. (F) MGDG and DGDG, produced in the envelopes by monogalactosyldiacylglycerol synthase 1 (MGD1) and digalactosyldiacylglycerol synthase 1 (DGD1), are the primary plastid lipids, and have crucial and disparate roles in PLB formation and etioplast-to-chloroplast transition, but more research is required to understand the role of both lipids and proteins in membrane biogenesis.

The response to light was one of the earliest phenomena observed in plants by naturalists, and much progress has been made in understanding both the perception of light by various photoreceptors, and the resultant signaling cascades that lead to transcriptional activation or repression of genes involved in de-etiolation. We will not discuss these processes, which have been recently reviewed (Casal *et al.*, 2014; Huang *et al.*, 2014; Casal and Qüesta, 2018; Pham *et al.*, 2018; Podolec and Ulm, 2018), but rather focus here on breakthroughs in post-transcriptional regulation and ultrastructural changes during etiolation and de-etiolation (summarized in Box 1; Fig. 2).

#### Small RNAs fine-tune temporal and spatial expression of genes during de-etiolation

Small regulatory RNAs (sRNAs) are 20–24 nt-long molecules that regulate gene expression via RNA-dependent DNA methylation, translation inhibition, or mRNA cleavage (reviewed in Borges and Martienssen, 2015; Singh *et al.*, 2018). Several important studies have highlighted the control of canonical light reception and response pathway factors by sRNAs, and the reciprocal light-based regulation not just of certain sRNA, but of the sRNA biogenesis process itself via these factors (Sorin *et al.*, 2005; Zhang *et al.*, 2011; Cho *et al.*, 2014; Tsai *et al.*, 2014; Achkar *et al.*, 2018; Sun *et al.*, 2018*b*). We refer the reader to two recent reviews (Sánchez-Retuerta *et al.*, 2018; Manavella *et al.*, 2019) for more details.

Recently, sRNAs were implicated in defining seedling tissue- or position- dependent greening responses: differential accumulation of certain sRNAs, and certain groups of sRNAs, was observed in different tissue types (Li *et al.*, 2014). Most recently, two large-scale studies were undertaken: Lin *et al.* (2017) profiled sRNAs during Arabidopsis de-etiolation, while Xu and colleagues (2017) undertook comparative miRNA profiling in rice and maize (*Zea mays*) to understand the establishment of photosynthesis in C_3_ versus C_4_ species. These studies, which defined several specific sRNA roles, such as the repression of photomorphogenic growth by miR396 via members of the Growth Regulating Factors family (Lin *et al.*, 2017), provide important pioneer work that defines global sRNA responses to greening (Fig. 2B). Furthermore, they demonstrate the use of de-etiolating systems—in which large scale yet highly temporally controlled changes occur—as a powerful tool for investigating pairwise relationships, for example, between regulators and their targets (Xu *et al.*, 2016; Page *et al.*, 2017; Xu *et al.*, 2017).

#### TOR connects light and nutrient signaling to activate translation

Target of rapamycin (TOR) is an evolutionarily conserved protein kinase that acts as a central hub to control cellular- and organism-level development (reviewed in Caldana *et al.*, 2019; Xiong and Sheen, 2014). Disruption of TOR results in plants with reduced chloroplast size and number, poorly developed thylakoid membranes, and decreased expression of key photosynthesis-related proteins (Xiong *et al.*, 2017). Furthermore, TOR (i) is required for proper regulation of photomorphogenic growth via regulation of translation and brassinosteroid signaling (Xiong *et al.*, 2017), (ii) acts as an indirect positive regulator of chlorophyll biosynthesis and photosynthesis-related genes (Li *et al.*, 2015), and (iii) is involved in the accumulation of the MGDG and DGDG synthases (Sun *et al.*, 2016). Thus, TOR positively contributes to plastid development. Nonetheless, seedlings with repressed TOR activity were recently reported to undergo more rapid accumulation of chlorophyll, PS-related transcripts, and plastid membrane lipids during de-etiolation—surprising results that the authors attributed to altered nutrient content of TOR-repressed seeds (Zhang *et al.*, 2018). Indeed, recent research underlines the essential role of TOR in sugar-status response during early development. This includes (indirect) positive control of cell elongation in dark-grown seedlings (Zhang *et al.*, 2016), and de-repression of shoot apical meristem growth in the dark via sugar-induced TOR activity (Li *et al.*, 2017*b*; Mohammed *et al.*, 2018). In light of a recently clarified position for TOR in the constitutively photomorphogenic 1 (COP1)–auxin cascade (Chen *et al.*, 2018*a*), these findings suggest that TOR balances light and sugar signaling to control plant and plastid development both at near-instantaneous and at more gradual time scales (Fig. 2C).

Recent studies have suggested that chloroplast protein production represents ~70% of the ATP cost of total cellular protein synthesis (Li *et al.*, 2017*a*), and two-thirds of the cellular nitrogen budget (Evans and Clarke, 2019). The need for greening seedlings to balance the cost of photosynthesis with its ultimate reward may therefore define (i) the control of gene expression that exerts control primarily at the (costly) translational stage (Shen *et al.*, 2009; Ning *et al.*, 2016); and (ii) the recently observed multi-phase accumulation of photosynthesis-related products and activities (Dubreuil *et al.*, 2018; Armarego-Marriott *et al.*, 2019). We note that, in addition to defining greening, the availability of resources like carbon (Kósa *et al.*, 2015) and nitrogen (Vitányi *et al.*, 2013) influences etioplast formation. Therefore, these recent works highlight the importance of considering resource availability in studying all aspects of etiolation and de-etiolation. Given that these resources arise from both (exhaustible) seed storage tissues and medium supplementation, it is clear that the choice of experimental system can largely influence observations.

#### Control by location: where is as important as when

As well as massive transcriptional changes (Ma *et al.*, 2001), greening can result in a global 2-fold increase in translational activity, and altered translation of ~1/3 of all transcripts (Liu *et al.*, 2012). Translation of cytosolic mRNAs can increase due to changes in the number of ribosomes on individual transcripts (ribosome density) or changes in the proportion of transcripts occupied by ribosomes (ribosome occupancy) (Liu *et al.*, 2013). In the plastid, transcripts are sequestered to membrane fractions in a ribosome-dependent manner, but membrane association of transcripts changes only minimally during maize leaf greening, suggesting that ribosome density, and not occupancy, drives greening-induced translation (Legen and Schmitz-Linneweber, 2017) (Fig. 2D1).

Within the cytosol, light-stimulated translation has been linked to processing bodies (P-bodies): RNA–protein complexes that are conserved in eukaryotes and regulate gene expression by degradation or translational arrest of mRNA (reviewed in Xu and Chua, 2011; Maldonado-Bonilla, 2014). Dark-grown seedlings of a P-body defective mutant (Xu and Chua, 2009) displayed prematurely opened apical hooks and augmented translation of thousands of transcripts, including those involved in the chlorophyll biosynthesis pathway (Jang *et al.*, 2019). Despite previous links between sRNA-mediated mRNA cleavage and P-bodies (Pomeranz *et al.*, 2010), Jang *et al.* (2019) noted limited overlap between mRNA cleavage and sequestration-induced translational ‘pausing’ (Fig. 2D2). Recently, physical sequestration has also been implicated in post-translational regulation. Localization of glutamyl-tRNA reductase (GluTR) to the chloroplast stroma, but not to the membrane, was associated with its enzymatic activity, and was shown to correlate with accumulation of chlorophyll during the early hours of greening (Schmied *et al.*, 2018). Interestingly, GluTR partitioning also changes following dark exposure of light-grown plants, suggesting that this regulation has relevance beyond the etioplast-to-chloroplast transition (Schmied *et al.*, 2018) (Fig. 2D3). Together, these recent studies underline that, in addition to cellular abundance of proteins and mRNAs, subcellular localization also needs to be taken into consideration.

#### Retrograde signaling: coupling the import and assembly of photosystems

Communication from the chloroplast to the nucleus, known as retrograde signaling, is a critical step during chloroplast biogenesis and maintenance (reviewed in Hernández-Verdeja and Strand, 2018; Rochaix and Ramundo, 2018; Leister, 2019; Pesaresi and Kim, 2019). Of six early identified *Genomes uncoupled* (*gun*) mutants defective in plastid-to-nucleus retrograde signaling (Susek *et al.*, 1993), five (*gun*2–6) have defects in genes for enzymes involved in tetrapyrrole biosynthesis. More recently, a role for the enigmatic GUN1 in regulating protein import via the cytosolic heat shock protein 90 (HSP90) chaperone was clarified using a de-etiolation system (Wu *et al.*, 2019). This followed observations that the GUN1 protein accumulates primarily during early chloroplast development (Wu *et al.*, 2018) and that *gun1* mutants showed retarded de-etiolation (Mochizuki *et al.*, 1996). The early flowering phenotype observed in *GUN1* overexpressing plants has led to the proposal that the protein may play a role in developmental phase transitions beyond chloroplast biogenesis (Wu *et al.*, 2018).

Singlet oxygen (^1^O_2_) is produced early during greening as a by-product of tetrapyrrole biosynthesis (Zhang *et al.*, 2015; Wang and Apel, 2019) and via early photosystem II (PSII) oxygen evolving complex activity (Zavafer *et al.*, 2015). In addition to potentially causing significant harm to the developing chloroplast, including damage to emerging PSII complexes prior to their protective incorporation into grana stacks (Shevela *et al.*, 2019), singlet oxygen may act in retrograde signaling via the Filamentation temperature sensitive H (FtsH; a membrane metalloprotease)-activated EXECUTER1 (EX1) pathway (Dogra *et al.*, 2017). Previous research suggests that the ^1^O_2_-mediated EXECUTER pathway primes etioplasts to develop into chloroplasts (Kim *et al.*, 2009), and also mediates high-light responses in the chloroplast, by regulation of multiple nucleus-encoded stress related transcripts (Carmody *et al.*, 2016). Localization of EXECUTER proteins to grana margins (Wang *et al.*, 2016*b*) further supports a potential role during PSII repair. Recently, Dogra *et al.* (2019) showed that the oxidation of a specific tryptophan residue (Trp643) in the singlet oxygen sensor domain contained in EX1 is essential for membrane localization and protein stability, and is also required for FtsH2-mediated EX1 degradation and further, as yet undefined, signaling to the nucleus (Dogra *et al.*, 2019). Interestingly, EX1 is also involved in carbon/nitrogen partitioning during light acclimation (Uberegui *et al.*, 2015), supporting a strong link between nutrient regulation and controlled chloroplast development (Fig. 2E).

#### Structural and functional membrane dynamics: recent focus on lipids in the regulation of membrane rearrangements

Although thylakoid membranes and etioplast internal membranes are both primarily composed of the galactolipids MGDG and DGDG, the lipid to lipid ratios (MGDG:DGDG) and lipid to protein ratios change with greening (Selstam and Sandelius, 1984). The role of lipid composition and content in plastid membrane structure has been studied extensively for several decades, but has recently returned to the spotlight with the publication of several studies involving disruption of galactolipid synthesis enzymes. Studies with mutants having slight decreases in galactolipid content and showing disrupted membranes in fully developed chloroplasts (Mazur *et al.*, 2019) display limited or no structural disruptions in etioplasts (Jarvis *et al.*, 2000), an effect attributable to the lower absolute requirement for lipids in etioplasts (Fujii *et al.*, 2014, 2017). In recent work, etiolated plants with severe MGDG and DGDG deficits were shown to accumulate less photoactive Pchlide, LPOR, and carotenoids compared with respective wild types (Fujii *et al.*, 2017, 2018). The decrease in photoactive Pchlide levels in a MGDG-deficient mutant observed under sugar-supplemented growth conditions (Fujii *et al.*, 2017) contrasts with previous Pchlide increases seen in soil-grown mutants (Aronsson *et al.*, 2008), again underlining the role of resource availability on plastid development. The decrease in DGDG content also resulted in significant structural PLB lattice perturbations, strong reduction of prothylakoid number, and retarded PLB disassembly in the light (Fujii *et al.*, 2019). Furthermore, while MGDG- and DGDG-deficient plants showed impairment in accumulation of Chl and the light-harvesting complex II protein LHCB1 during greening, changes in photosynthesis-related gene transcript accumulation were, relatively, delayed (Fujii *et al.*, 2019), suggesting that lipid status is sensed indirectly (e.g. via disrupted protein insertion or function).

While these studies suggest differences in the roles of MGDG and DGDG during etiolation and de-etiolation, it is difficult to make concrete conclusions, due to the different reduction of galactolipid contents in each mutant and the inter-relationship between the lipids (DGDG is a downstream product of MGDG). These issues argue for alternative systems, such as the *in vitro* system recently used to show the requirement for MGDG and charged lipids in regulating LPOR complex formation and activity (Gabruk *et al.*, 2017), and support a need for further biophysical studies that investigate the detailed distribution of lipid phases inside membranes (Garab *et al.*, 2017; Ughy *et al.*, 2019). *In vivo* time-resolved 3D techniques (e.g. Kowalewska *et al.* 2016), may be used to answer several open questions in the field, including how the PLB is formed and how and from where membrane components are recruited during the formation of grana stacks. On the latter topic, inner membrane-localized MGDG synthase has been suggested to be both a point of contact between thylakoids and the inner envelope membrane, and a supplier of lipids during thylakoid biogenesis (Rocha *et al.*, 2018). We note that the nature of contact point(s), as being either direct or involving vesicles or tubules, remains debated (reviewed in Lindquist *et al.*, 2016; Lindquist and Aronsson, 2018; Mechela *et al.*, 2019). Notably, a recent 3D analysis of the proplastid-to-chloroplast transition (Liang *et al.*, 2018) visualized direct connection points, which were proposed to both act as lipid transfer points and align growing thylakoids. Given that factors associated with these connections have been implicated in both thylakoid biogenesis and maintenance (e.g. Gao *et al.*, 2006; Patil *et al.*, 2018), understanding such connections is likely to bear importance throughout the lifetime of the plastids (Fig. 2F).

### Etiolation studies and the future

#### New systems required and new lessons learned

**Fig. 3.**
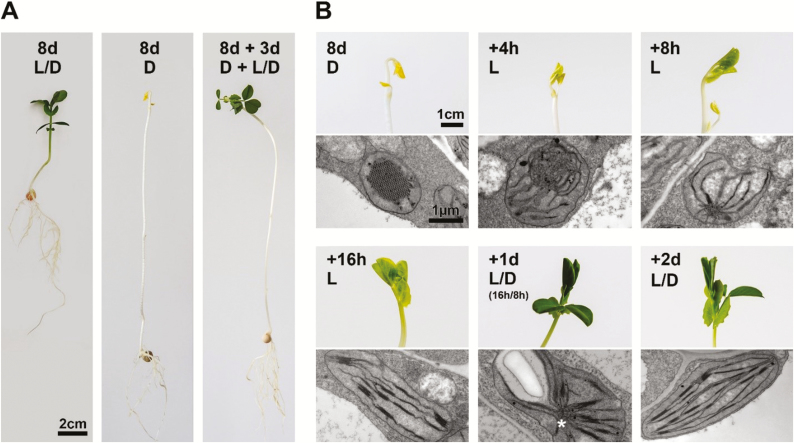
Pea (*Pisum sativum*) de-etiolating under light/dark conditions. (A) Pea seedlings grown for 8 d under light dark (L/D) conditions (16 h of light at 40 µmol photons m^−2^ s^−1^–8 h of darkness) (left panel), darkness (D) (middle panel), 8 d of darkness followed by 3 d of L/D (right panel). Pea, which develops true leaves in darkness, as well as other hypogeal germinating plants, may be used as an alternative system to epigeal germinating Arabidopsis plants, which only develop cotyledons in the dark. As ‘maternal tissue’, cotyledons are formed by and undergo different developmental programing from true leaves. (B) De-etiolating pea. The upper panels show seedling shoot apices; the lower panels show transmission electron micrograph. Plants grown in darkness for 8 d were de-etiolated under light–dark conditions (16 h of light at 40 µmol photons m^−2^ s^−1^–8 h of darkness). Note that following the first 24 h of growth there is partial reformation of the PLB, indicated by the white asterisk.

To date, etiolation and de-etiolation work focused on the study of molecular processes has commonly been undertaken with dark-grown seedlings. The benefits of this system include that it (i) requires limited growth time and space yet provides sufficient material compared with other experimental systems such as the shoot apical meristem, and (ii) is highly customizable by use of different timing and lighting regimes and introduction of different substances to the growth medium (López-Juez *et al.*, 2008; Mohammed *et al.*, 2018; Dóczi *et al.*, 2019). Nonetheless, there are limitations to this system, which should not be overlooked. These include the difficulties in separating plastid development (i.e. etioplast-to-chloroplast transition) from general seedling development programs, as well as issues associated with observing chloroplast development only in cotyledons, which are programmed differently from true leaves (reviewed in Pogson *et al.*, 2015). Some limitations of the present system may be overcome by using other species and systems, although we stress that both etioplast formation and light-induced de-etiolation may largely differ depending on the species, timing, and conditions used (Skupień *et al.*, 2017), making cross-system comparisons difficult. For example, both runner bean (*Phaseolus coccineus*) and pea (*Pisum sativum*) (Kowalewska *et al.*, 2016) show similar skotomorphogenic growth to Arabidopsis, yet develop true leaves in darkness (Fig. 3). PLBs have also been observed in non-seedling systems, both in young leaves of tobacco following extended dark treatment (Armarego-Marriott *et al.*, 2019) and in the innermost leaf primordia of the closed and opening leaf buds of trees (Solymosi and Böddi, 2006; Solymosi *et al.*, 2006, 2012). The problem of uneven lighting that arises from gradual cotyledon opening or seed-coat shading (e.g. Solymosi *et al.*, 2007) was recently overcome by using duckweed (*Landoltia punctate*), a flat-leafed aquatic monocot (Monselise *et al.*, 2015). More artificially, cell cultures (Dubreuil *et al.*, 2018), and even a callus-based system (Schaub *et al.*, 2018), have been used to investigate various aspects of plastid development, and may putatively be adapted for de-etiolation. Nonetheless, these experimental systems come with their own caveats, in particular multiple impacts of carbon supplementation on plastid development (Eckstein *et al.*, 2012; Häusler *et al.*, 2014). Such systems may help to address issues related to spatial diversity of plastid types, seen previously within the shoot apical meristem (Charuvi *et al.*, 2012), in chloroplasts in different leaf regions (Gügel and Soll, 2017), and in etioplasts within different tissues (Kósa *et al.*, 2015) or even single cells (Solymosi *et al.*, 2012).

Curiously, while the etiolated state is largely defined by both the presence of a paracrystalline PLB and the absence of (stacked) thylakoid membranes, early studies in cucumber (*Cucumis sativus*; Ikeda, 1970) and avocado (*Persea americana*; Cran and Possingham, 1973), and more recent findings in bean (*Phaseolus vulgaris*) (Schoefs and Franck, 2008), various tree species (Solymosi *et al.*, 2006), and tobacco (*Nicotiana tabacum*) (Armarego-Marriott *et al.*, 2019), demonstrate that both structures can co-exist in a single plastid. Indeed, several studies indicate that PLB reformation may occur in young chloroplasts during extended darkness, or even during normal night periods during de-etiolation (see Fig. 3; Rudowska *et al.*, 2012; Skupień *et al.*, 2017; reviewed in Solymosi and Aronsson, 2013). These findings underscore the important influence of light regime, as well as light intensity, quality, and circadian-related effects (reviewed in Seluzicki *et al.*, 2017) on greening, factors that must be considered when observing plastid development. We suggest PLB reformation as an interesting field for future study, and underline that the use of diverse systems may both further clarify current understandings of PLB formation and dissolution, and suggest new directions for future works.

#### Using etiolated systems and knowledge to go ‘beyond the darkness’

The benefits of the standard seedling etiolation and/or de-etiolation systems means that they have been used often in recent years to study diverse topics including gravitropism (Yamamoto *et al.*, 2017), phototropism (Sullivan *et al.*, 2019), resource limitation (Avin-Wittenberg *et al.*, 2015; Kósa *et al.*, 2015), and metabolite or hormone signaling (Gupta *et al.*, 2015). Furthermore, etiolated growth can promote development of (i) certain tissue and organ types (e.g. adventitious roots; Sorin *et al.*, 2005; da Costa *et al.*, 2018; Trinh *et al.*, 2018), (ii) certain growth types (e.g. growth by cellular expansion in hypocotyls; Sinclair *et al.*, 2017; Ilias *et al.*, 2019), and (iii) specific responses (e.g. ethylene ‘triple response’; Guzmán and Ecker, 1990; Ma *et al.*, 2018) that cannot be easily observed in light-grown plants. Growth in darkness can also induce arrest of the shoot apical meristem, and thus de-etiolation can be used to observe shoot apical meristem development (López-Juez *et al.*, 2008; Mohammed *et al.*, 2018; Dóczi *et al.*, 2019).

Beyond the practicality of the system itself, the greatest value of etiolation/de-etiolation studies lies in the central role of light signaling in plant life. Indeed, the overlap between factors involved in light responses with those involved in other response and growth processes has allowed basic knowledge from etiolation studies to be used to understand diverse plant processes (reviewed in Liu *et al.*, 2017; Hsieh and Okamoto, 2014; Casal and Qüesta, 2018). In the applied sector, associations have been made between light receptors or responses and desirable crop attributes such as dwarfism (Hou *et al.*, 2017), fruit or flower chromoplast development (Pankratov *et al.*, 2016), and abiotic stress response (Zhou *et al.*, 2018). Shade avoidance responses bear similarity to etiolation (Wang *et al.*, 2016*a*), while ‘photobiotechnology’, in which modulated expression results in improved crop yield and resistance, has recently been proposed for improved food security (Ganesan *et al.*, 2017). Clearly, future attempts to improve photosynthesis will require a detailed understanding of the chloroplast membrane structures and their biogenesis, as well as a thorough understanding of the processes involved in regulating the expression of photosynthesis-related genes (Ort *et al.*, 2015). Taken together, while there is still much more to be learnt about de-etiolation itself, it is also clear that etiolation and de-etiolation systems provide the ideal environments to gain insight into the establishment of one of the most important processes for plant growth.

Box 1. **Key developments in understanding de-etiolation**

**Small regulatory RNAs are highly dynamic during greening**

Recent large-scale studies of small regulatory RNA (sRNA) changes during greening in Arabidopsis (Lin *et al.*, 2017), rice, and maize (Xu *et al.*, 2017) provide pioneer datasets, suggest new roles for several sRNAs, and demonstrate the power of de-etiolation systems in investigating pairwise relationships.

**TOR connects light and nutrient signaling**

The indirect activator of translation, target of rapamycin (TOR), acts downstream of the COP1–auxin cascade during de-etiolation (Chen *et al.*, 2018*a*), but is also involved in light-independent developmental regulation in response to sugars (Mohammed *et al.*, 2018). The complex demand/supply of resources associated with establishing photosynthesis has implications for the regulation and kinetics of chloroplast development, and for currently used etiolation systems.

**Availability, not just abundance, counts for transcripts and proteins**

Thousands of mRNA species are present yet translationally repressed by sequestration to processing bodies (P-bodies) in the dark (Jang *et al.*, 2019). For plastid-encoded thylakoid membrane proteins, association of respective mRNA to ribosomes localizes them to membranes, but the membrane to soluble mRNA fraction changes little during greening (Legen and Schmitz-Linneweber, 2017). Soluble versus membrane localization of glutamyl-tRNA reductase (GluTR) does change with lighting, and the soluble (active) fraction shows early correlation with chlorophyll content (Schmied *et al.*, 2018).

**Singlet oxygen causes PSII damage and acts as a retrograde signal during de-etiolation**

The early assembly of the PSII oxygen evolving complex results in the (damaging) formation of singlet oxygen (^1^O_2_; Shevela *et al.*, 2019). ^1^O_2_ retrograde signaling mediates de-etiolation via the EXECUTER1 pathway (Chen *et al.*, 2015; Carmody *et al.*, 2016). A de-etiolation system was recently used to assign function to the elusive integrator of retrograde signalling, GUN1 (Wu *et al.*, 2018).

**Finally looking at membrane lipids (and how they get there)**

Three recent studies investigated the effect of decreased MGDG (Fujii *et al.*, 2017) and DGDG (Fujii *et al.*, 2018) content on etioplast formation and greening (Fujii *et al.*, 2019). They emphasize the role of DGDG in the dynamics of tubular-lamellar transformation occurring during PLB–thylakoid membrane transition as well as the crucial role of both neutral galactolipids in the membrane-associated steps of Chl biosynthesis. Future studies, using diverse systems and 3D imaging techniques, are suggested to further this developing field.

## Abbreviations:

COP1: constitutively photomorphogenic 1
DGDG: digalactosyldiacylglycerol
EX1: Executer 1
FtsH: Filamentation temperature sensitive H
GluTR: Glutamyl-tRNA reductase
GUN1: Genomes Uncoupled 1
HSP90: heat shock protein 90 (chaperone)
LPOR: light dependent protochlorophyllide oxidoreductase
MGDG: monogalactosyldiacylglycerol
Pchlide: protochlorophyllide
PLB: prolamellar body
PSII: photosystem II
P-bodies: processing bodies
sRNA: small RNA
TOR: target of rapamycin
